# Sequence and Haplotypes Variation of the Ovine Uncoupling Protein-1 Gene (*UCP1*) and Their Association with Growth and Carcass Traits in New Zealand Romney Lambs

**DOI:** 10.3390/genes9040189

**Published:** 2018-03-30

**Authors:** Qingming An, Huitong Zhou, Jiang Hu, Yuzhu Luo, Jon G. H. Hickford

**Affiliations:** 1 Gansu Key Laboratory of Herbivorous Animal Biotechnology, Faculty of Animal Science and Technology, Gansu Agricultural University, Lanzhou 730070, China; anqingming2009@163.com (Q.A.); Zhou@lincoln.ac.nz (H.Z.); huj@gsau.edu.cn (J.H.); 2 Faculty of Wujiang, Tongren University, Tongren 554300, China; 3 Gene-Marker Laboratory, Faculty of Agriculture and Life Sciences, P.O. Box 84, Lincoln University, Lincoln 7646, New Zealand

**Keywords:** uncoupling protein-1 gene (*UCP1*), variation, haplotype, carcass traits, sheep

## Abstract

Uncoupling protein-1 gene (*UCP1*) plays an important role in the regulation of thermogenesis, energy expenditure, and protection against oxidative stress. In this study, six separate *UCP1* regions: region-1 and region-2 (two parts of the promoter), region-3 and region-4 (two parts of intron 1), region-5 (spanning part of intron 5 and part of exon 6), and region-6 (spanning part of exon 6 and part of the 3′-UTR) from a variety of sheep breeds, were analysed using polymerase chain reaction-single-stranded conformational polymorphism (PCR-SSCP) analyses. In total, 30 single nucleotide polymorphisms (SNPs) were detected. Of these, 14 were located in the promoter, eight were found in intron 1, six were found in intron 5, and one was found in the 3′-UTR. One substitution in exon 6 (c.910A/G) would putatively result in an amino acid change (p.Thr304Ala). Twenty-eight novel SNPs and nine new haplotypes spanning region-2 to region-5 were identified. Of these nine haplotypes, five were common (*B*_2_*-A*_5_, *C*_2_*-A*_5_, *C*_2_*-C*_5_, *A*_2_*-A*_5_, and *A*_2_*-B*_5_) and four were rare (*C*_2_*-B*_5_, *A*_2_*-C*_5_, *B*_2_*-C*_5_, and *B*_2_*-B*_5_) in the sheep investigated. Of the five common haplotypes found in 314 New Zealand Romney sheep for which growth and carcass trait data were available, the presence of *A*_2_*-B*_5_ was associated with decreased hot carcass weight (HCW) and loin lean-meat yield (*p* = 0.006, *p* = 0.032, respectively), and the presence of *C*_2_*-C*_5_ was associated with a decreased proportion of leg lean-meat yield (*p* = 0.047) in the carcasses. No associations were found with growth traits. These results confirm that ovine *UCP1* is a variable gene and may have value as a genetic marker for sheep breeding.

## 1. Introduction

Uncoupling protein-1 (UCP1) is a member of the uncoupling protein (UCP) family and belongs to a super-family of carrier proteins that are typically located in the inner membrane of mitochondria. In mammals, the UCP family contains at least six members, including UCP1, UCP2, UCP3, UCP4, UCP5 or BMCP1 (brain mitochondrial carrier protein-1), and UCP6 [1,2]. These proteins play pivotal roles in the regulation of thermogenesis, in regulating energy expenditure, and in providing protection against oxidative stress [3,4].

The UCP proteins have similar molecular structures, but varying levels of expression in different tissues, including brown adipose tissue, white adipose tissue, skeletal muscle, thymocytes, and the liver [5,6,7,8]. Among the UCP proteins, UCP1 is predominantly found in brown adipose tissue [9], but recently it has been reported to be found in many other tissues, including white adipose tissue, skeletal muscle, longitudinal smooth muscle layers, thymocytes, and the pancreatic islets [10].

The UCP1 gene (*UCP1*) and its expression have been studied in humans and mice [9,10]. Genetic variation has been detected in some species, and this variation has been found to be associated with fat metabolism, type 2 diabetes risk, and obesity [10,11]. There are very few reports describing polymorphism in *UCP1* in livestock, but recently there have been two reports of *UCP1* variation in sheep [12,13]. Yang et al. described variation in three regions (the promoter, intron 2, and exon 5) of the gene, revealing three promoter region variants (designated *A*, *B*, and *C*) and two intron 2 variants (*a* and *b*) [13]. The presence of *B* in a lamb’s genotype was associated with decreased subcutaneous fat depth and proportion of total lean-meat yield of loin meat, and an increased proportion of total lean-meat yield of hind-leg meat. In contrast, having two copies of *C* was associated with increased subcutaneous carcass fat depth and proportion of total lean-meat yield of shoulder meat, and a decreased hind-leg yield. Yuan et al. described ten single nucleotide polymorphisms (SNPs) in the exon 2, exon 5, and 3′-UTR regions, and the variation appeared to affect mRNA levels in different tissues [13].

*UCP1* has also been identified in fish and cattle, and across these species, and humans and mice; it appears to have a conserved structure of six exons interrupted by five introns. In the species studied to date, the gene spans approximately 6.7 kb. Analysis and comparison of the various *UCP1* sequences deposited in GenBank and Ensembl suggest that further unpublished sequence variation exists, although this may also reflect sequencing errors.

In this study, the objective was to use polymerase chain reaction single-stranded conformational polymorphism (PCR-SSCP) analysis to search for and confirm the presence of SNPs in *UCP1* in various sheep breeds. This method allows for the large-scale screening of variation in PCR amplicons of size up to approximately 600 base-pairs (bp), and it is sensitive to the level of detecting single nucleotide variation (i.e., SNPs). The method was then employed to investigate associations between ovine *UCP1* haplotypes and various production traits in New Zealand (NZ) Romney lambs.

## 2. Materials and Methods

All research involving animals was carried out in accordance with the Animal Welfare Act 1999 (New Zealand Government) and the collection of sheep blood drops by nicking sheep ears is covered by Section 7.5 Animal Identification of the Animal Welfare (Sheep and Beef Cattle) Code of Welfare 2010, which is a code of welfare issued under the Animal Welfare Act 1999 (New Zealand Government).

### 2.1. Sheep Investigated and DNA Collection

Sheep (*n* = 311) selected from a variety of breeds that are found in NZ and China, including Merino (*n* = 50), Dorset Down (*n* = 22), Dorper (*n* = 20), Suffolk (*n* = 12), NZ Romney (*n* = 97), Corriedale (*n* = 13), Perendale (*n* = 19), cross-bred sheep (*n* = 28), and Chinese Tibetan sheep (*n* = 50), were used to screen for variation in *UCP1* using methods that are described below.

Having identified that variation existed in these sheep, an additional 1185 NZ Romney lambs from 17 independent sire-lines (rams sourced from farms spread across the South Island of NZ), but born and raised on one farm, were investigated to ascertain if associations exist between ovine *UCP1* haplotypes and production traits.

All of the lambs were ear-tagged with a unique identification number within 12 h of birth, and their birth date, birth weight, birth rank (whether the lamb was born single, twin, or triplet), and gender were recorded. All of the lambs were tailed at approximately three weeks of age and weighed at that time. At weaning, a blood sample from the lambs was collected onto an FTA card (Whatman BioScience, Middlesex, UK).

Weaning occurred at approximately three months of age, and all of the lambs were weighed. Their pre-weaning growth rate was calculated as the difference between weaning weight and birth weight, divided by their individual age in days (expressed in grams/day).

At weaning, female lambs were drafted off and kept as flock replacements and male lambs weighing 36 kg and over were drafted and sent to the Alliance Group Limited meat processing plant at Pukeuri, NZ, for slaughter. Those ram lambs under 36 kg live weight were retained on pasture for another four weeks. At the second draft, lambs were again drafted for slaughter at weights of 36 kg and over. The third and final draft consisted of all the remaining male lambs regardless of their weight. Draft age and draft weight were recorded for each male lamb.

Hot carcass weight (HCW) was measured on the lambs directly at slaughter. HCW is the weight in kilograms of the carcass minus the pelt, head, feet, and gut. Other carcass composition data were subsequently collected using video imaging analysis (VIAScan^®^ Sastek, Hamilton, Australia), including carcass fat depth at the 12th rib (V-GR), loin lean-meat yield, shoulder lean-meat yield, leg lean-meat yield, and total lean-meat yield (the sum of the individual leg, loin, and shoulder lean-meat yields for any given carcass), and all recorded as a percentage of HCW. The proportion yield of leg, loin, or shoulder was also recorded, this being the yield of the specific part of the carcass divided by the total yield and expressed as a percentage. It describes the distribution of lean meat on the carcass.

Of the 1185 lambs originally born and for which blood was collected, growth and genotype data were subsequently collected for 1083 lambs. Of these, 456 male lambs were slaughtered, but as a consequence of the data being incomplete for all traits for some lambs, only 314 were used in the association analyses.

### 2.2. Polymerase Chain Reaction Amplification and Single-Stranded Conformational Polymorphism Analysis

**Figure 1:**
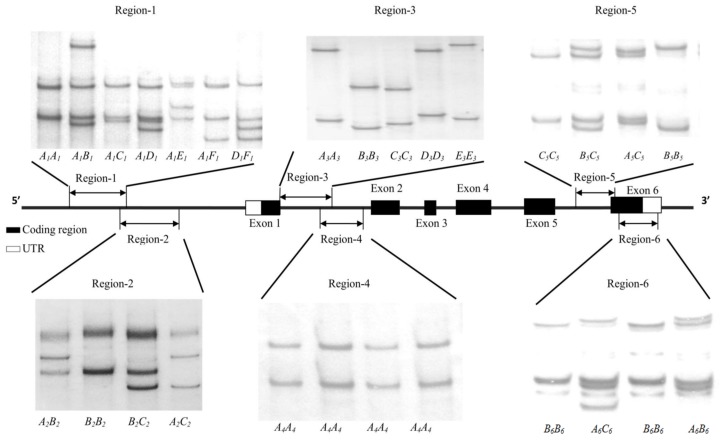
Polymerase chain reaction-single-stranded conformational polymorphism (PCR-SSCP) banding patterns of ovine uncoupling protein-1 gene (*UCP1*). Unique PCR-SSCP patterns representing different DNA sequences were detected. Six unique patterns corresponding to six variants sequences (*A*_1_*-F*_1_) were found for region-1; three unique patterns corresponding to three variants sequences (*A*_2_*-C*_2_, *A*_5_*-C*_5_, and *A*_6_*-C*_6_) in region-2, region-5, and region-6, respectively; five unique patterns corresponding to five variants sequences (*A*_3_*-E*_3_) in region-3; pattern corresponding to one variant in region-4.

DNA for analysis was purified from the FTA cards using a procedure described by Zhou et al. [14]. Six different regions of ovine *UCP1* were chosen for analysis (see Figure 1). These were region-1 and region-2 (two separate parts of the promoter), region-3 and region-4 (two separate parts of intron 1), region-5 (spanning part of intron 5 and part of exon 6), and region-6 (spanning part of exon 6 and part of the 3′-UTR). Six sets of PCR primers were designed to amplify these regions, and their sequences and coordinates relative to JN604985.1 are described in Table 1. These primers were synthesized by Integrated DNA Technologies (Coralville, IA, USA).

Amplifications were performed in a 15-µL reaction containing the purified genomic DNA on one punch of the FTA card, 0.25 µM of each primer, 150 µM of each dNTP (Bioline, London, UK), 2.5 mM of Mg^2+^, 0.5 U of Taq DNA polymerase (Qiagen, Hilden, Germany), and 1× reaction buffer supplied with the enzyme. The thermal profile for the regions amplified consisted of an initial denaturation for 2 min at 94 °C followed by 35 cycles of 30 s at 94 °C, 30 s at the annealing temperatures shown in Table 1, and 30 s at 72 °C with a final extension of 5 min at 72 °C. Amplification was carried out in S1000 thermal cyclers (Bio-Rad, Hercules, CA, USA).

Amplicons were then subjected to SSCP analysis to screen for variation. In these analyses, a 0.7-µL aliquot of each amplicon was mixed with 7 µL of loading dye (98% formamide, 10 mM EDTA (Ethylenediaminetetraacetic acid), 0.025% bromophenol blue, 0.025% xylene-cyanol), and after denaturation at 95 °C for 5 min the samples were cooled rapidly on wet ice and loaded on to 16 cm × 18 cm, 12% or 14% acrylamide:bisacrylamide (37.5:1) (Bio-Rad) gels. Electrophoresis was performed using Protean II xi cells (Bio-Rad) for 19 h in 0.5 × TBE (Tris/Borate/EDTA) and the conditions described in Table 1. The gels were silver-stained by the method of Byun et al. [15].

### 2.3. DNA Sequencing and Sequence Analyses

Amplicons representative of different SSCP patterns from sheep that appeared to be homozygous were sequenced directly at the Lincoln University DNA Sequencing Facility. Amplicons that were only found in heterozygous sheep were analysed using an approach that has been described previously [16]. Briefly, a band corresponding to the allele was excised as a slice from the polyacrylamide gel, macerated, and then used as a template for re-amplification with the original primers. This second amplicon was then sequenced directly.

Sequence alignments, translations, and comparisons were carried out using DNAMAN version 5.2.10 (Lynnon BioSoft, Vaudreuil, QC, Canada). The BLAST algorithm was used to search the NCBI GenBank databases for homologous sequences.

### 2.4. Haplotype Determination

Haplotypes were constructed across region-2 and region-5 of *UCP1*, as these two regions were found to have the most variants that were common (i.e., occurred at a frequency of over 10%). As these two regions amplified are nearly 8 kb apart and cannot easily be directly sequenced to detect the haplotype, we instead ascertained the haplotype using the following approach.

Progeny that typed as homozygous in either of the regions could directly have their haplotypes inferred based on the co-inheritance of sequences. For example, if a sheep presented with genotype *A_2_A_2_* in region-2 and genotype *A*_5_*B*_5_ in region-5, the presence of haplotypes *A*_2_-*A*_5_ and *A*_2_-*B*_5_ could be directly inferred.

If progeny typed as heterozygous in both regions, their haplotype could be inferred from their sire haplotype and a comparison with other progeny from the same sire. For example, for a sire having a diplotype of *A*_2_-*B*_5_*/A*_2_-*C*_5_, approximately half of its offspring will have the haplotype *A*_2_-*B*_5_, while the other half will have *A*_2_-*C*_5_.

### 2.5. Statistical Analyses

All statistical analyses were performed using Minitab version 16 (Minitab Inc., State College, PA, USA). Unless otherwise indicated, all *p* values were considered statistically significant when *p* < 0.05 and trends were noted when 0.05 ≤ *p* < 0.10.

General Linear Mixed Models (GLMMs) were used to assess the effect of the presence or absence of a particular haplotype (i.e., those with frequencies over 10%) on birth weight, weaning weight, growth rate to weaning, and the various carcass traits, including HCW, VIAScan fat score (V-GR), leg lean-meat yield, loin lean-meat yield, shoulder lean-meat yield, total carcass lean-meat yield, and proportion lean-meat yield of leg, loin, and shoulder.

For birth weight traits, birth rank (single, twin, and triplet), sire, and gender were fitted as fixed factors in the models. For the tailing weight and weaning weight traits, rearing rank (single, twin, and triplet) and gender were fitted as fixed factors, and sire and age were fitted as random factors in the models. For pre-weaning growth rate traits, rearing rank (single, twin, and triplet) and gender were fitted as fixed factors, and sire was fitted as a random factor in the models. For carcass traits, rearing rank (single, twin, and triplet) was fitted as a fixed factor, sire was fitted as a random factor in the models, and draft age was fitted as a covariate.

In the models, each haplotype was coded as either present (1) or absent (0) for each lamb analysed. The effects were first assessed using single-haplotype presence/absence models. For any trait for which more than one haplotype had an association with *p* < 0.200, and thus where another haplotype could potentially have an impact on the associations being described in the single-haplotype model, a second series of multi-haplotype models were performed, where these other haplotypes were also included as explanatory factors in the models.

## 3. Results

### 3.1. Variation Detected in Ovine Uncoupling Protein-1 Gene

Six PCR-SSCP patterns were observed for region-1 and three PCR-SSCP patterns were observed for each of region-2, region-5, and region-6. Five PCR-SSCP patterns were observed for region-3 and only one PCR-SSCP banding pattern was observed for region-4. After sequencing, these PCR-SSCP patterns were confirmed as novel variant sequences of ovine *UCP1* and the sequences (except for the region-2 sequences which have been reported previously [13] and the region-4 sequence in which no variation was not found) were deposited into GenBank with accession numbers as follows: *A_1_-F_1_*: KT161246-KT161251; *A*_3_-*E*_3_: KT161235-KT161239; *A*_5_-*C*_5_: KT161240-KT161242; and *A*_6_-*C*_6_: KT161243-KT161245. The PCR-SSCP banding patterns observed for the two fragments are illustrated in Figure 1.

A total of 30 SNPs were identified in the sheep breeds analysed (Table 2). Of these, 14 were located in the promoter region, eight in intron 1, six in intron 5, and one in the 3′-UTR. One substitution in exon 6 (c.910A/G) would potentially result in an amino acid change (p.Thr304Ala). Except for c.−1334T/A and c.−1299G/A, the other 28 SNPs are reported for the first time.

In the 311 sheep of various breeds studied, *A*_1_ (region-1) and *A*_3_ (region-3) were the most common variants, with overall frequencies of 86.6% and 90.2%, respectively. In region-6, *A*_6_ and *B*_6_ were the most common variants with frequencies of 50.0% and 44.3%, respectively, but *C*_6_ was rare (5.7%). In region-2, *A*_2_ was the most common variant with a frequency of 56.4%, and the *B*_2_ and *C*_2_ variants were next most common with overall frequencies of 26.2% and 17.5%, respectively. In region-5, *A*_5_ and *C*_5_ were the most common variants with frequencies of 41.3% and 45.7%, respectively.

Nine haplotypes that spanned region-2 to region-5 of ovine *UCP1* were identified in 861 NZ Romney sheep. Of these haplotypes, *B*_2_-*A*_5_, *C*_2_-*A*_5_, *C*_2_-*C*_5_, *A*_2_-*A*_5_, and *A*_2_*-B*_5_ were the most common. The other four haplotypes (*C*_2_-*B*_5_, *A*_2_-*C*_5_, *B*_2_-*C*_5_, and *B*_2_-*B*_5_) were rare, each occurring at a frequency of less than 10%.

### 3.2. Effect of Variation in UCP1 on Various Production Traits

In the single-haplotype (presence/absence) model, the presence of haplotype *A*_2_-*B*_5_ was associated with decreased HCW (*p* = 0.006) and loin lean-meat yield (*p* = 0.032) (Table 3). The presence of haplotype *C*_2_-*C*_5_ was associated with decreased proportion leg lean-meat yield (*p* = 0.047). These haplotype associations remained significant when the other haplotypes (where *p* < 0.200) were factored into the models. No associations were found between the other haplotypes and other carcass traits in the lambs studied (Table 3).

No association was found between ovine *UCP1* variation and birth weight, tailing weight, weaning weight, and pre-weaning growth rate in the NZ Romney lambs studied (results not shown).

## 4. Discussion

This investigation explored genetic variation in six different regions of ovine *UCP1* in a variety of sheep breeds from NZ and China. The different regions of the gene analysed included parts of the promoter, intron 1, intron 5, exon 6, and the 3′-UTR. These regions were chosen because they have previously been identified to be of importance in human, cattle, sheep, and chicken *UCP1* association studies [13,17,18,19]. A total of 30 SNPs were found (see Table 2), suggesting that the ovine *UCP1* is quite variable. In this regard, extensive heterogeneity has also been described in humans [17,20], rats [11,21], cattle [18], and chickens [19]. 

Substitution c.910 A/G was the only SNP identified in the coding sequence. This would potentially result in an amino acid change (p.Thr304Ala) at the C-terminal end of the third loop transmembrane domain [22]. It is therefore conceivable that this amino acid substitution may affect the transport of ATP/ADP and thus change the function of UCP1 during muscle growth.

We detected fourteen SNPs in the promoter regions analysed (see Table 2). The possible effect of these substitutions is difficult to ascertain in the context of not having undertaken functional studies. It is notable that between c.−1846 and c.−1538 in the rat *UCP1* promoter, multiple binding sites have been identified, including peroxisome proliferator-activated receptors (PPARs), a retinoic acid response element (RARE), a cAMP response element binding protein (CREB) binding site, and other enhancer elements. Knock-out studies in mice suggest that these binding sites and elements may have either negative or positive effects on the activity of the *UCP1* promoter, and hence affect the expression of *UCP1* in different tissues [21,23].

A total of 14 nucleotide substitutions were found in *UCP1* intron 1 and intron 5. Intron variation may not directly affect the structure of UCP1, but it can affect transcription efficiency by affecting regulatory elements, such as enhancers, silencers, or other DNA structures [24]. In addition, variation in introns may be linked to other variation in coding regions of the gene or regions that may affect the processing of the primary transcript or its stability. Together, the above findings support the conclusion that additional investigation of *UCP1* promoter and intron region variation is warranted.

Haplotypes can span a larger region of a gene and help increase the Polymorphism Information Content (PIC). They can therefore be used to improve the power of association studies [25]. Nine haplotypes were identified that spanned region-2 to region-5. These regions have been suggested to regulate *UCP1* function in humans, cattle, and chickens. For example, Mori et al. described A>C SNP in the 5’UTR of human *UCP1* and revealed that the C variant occurred more frequently in a group of type II diabetes patients than in a control group [17]. They also described p.Met229Leu, an amino acid change that was in linkage with the A>C SNP and that was also associated with the difference between the type II diabetes patients and the controls. Ferraz et al. [18] described a C>G SNP in intron 1 of bovine *UCP1* and it was revealed to be associated with HCW. Cattle with the GG genotype were 2.19 kg heavier than those with the CC genotype. The SNP was also associated with back fat (BF) thickness, and again, animals with the GG genotype had 0.28 mm more BF than those with the CC genotype. This supports our finding of an association between *UCP1* variation and HCW in sheep, but we did not find an effect on V-GR, the VIAScan fat measurement. This is perhaps unsurprising given the small effect seen in cattle.

The absence of haplotype *A*_2_-*B*_5_ was associated with an increase in HCW and loin lean-meat yield (0.77 ± 0.44% and 0.30 ± 0.21%, respectively), the values being estimated from the predicted means presented in Table 3. This suggests that the absence of haplotype *A*_2_-*B*_5_ would predict a higher slaughter weight and presence of more lean-meat. Although this improvement is small, it could nevertheless be of benefit in improving carcass composition traits.

In this study, no association was found between ovine *UCP1* variation and V-GR. This is inconsistent with a previous report suggesting that variants of ovine *UCP1* are associated with variation in this trait [13]. This inconsistency may be due to the different sheep studied or because the average V-GR was higher (6.6 ± 0.31 mm) for the sheep studied by Yang et al. [13] compared with 2.43 ± 0.59 mm in this study. The difference in V-GR would suggest that sheep had improved nutrition and thus laid down more subcutaneous fat, and this suggests that the role of *UCP1* in fat deposition may be complex.

In summary, the work presented in this study confirms that ovine *UCP1* has high levels of polymorphism, and it could therefore be speculated that even more new variants will be found when more samples and different sheep breeds are investigated. Given the apparently important role of *UCP1* in lipolysis and thermogenesis and reports from other species that *UCP1* variation is associated with a variety of phenotypes, further research into the role of *UCP1* in determining carcass traits seems warranted.

## Figures and Tables

**Table 1:** The primer sequences and single-stranded conformational polymorphism (SSCP) conditions for the analysis of regions of ovine uncoupling protein-1 gene (*UCP1*).

Region Amplified	Primer Binding Regions ^a^	Primer Sequence (5′-3′)	PCR Annealing Temperature	Predicted Amplicon Size	SSCP Condition
Region-1	517–535	F: TGGACGACAGGGAAGCCAG	60 °C	393 bp	200 V, 14%, 23.0 °C
	889–909	R: TATTCGCAGATTCCTCTCATC			
Region-2	853–873 ^b^	F: AGATACAAGCGGAAGAGACAC	62 °C	351 bp	300 V, 12%, 12.0 °C
	1185–1203 ^b^	R: TGAAGGGTTGGGTCTGTCA			
Region-3	2501–2519	F: CGCCAAAGTCCGGCTACAG	61 °C	304 bp	320 V, 14%, 20.0 °C
	2785–2804	R: CTAGGCAGTAGGCTAGGAAG			
Region-4	2748–2766	F: TGTCAACCTCTCCTGCACG	61 °C	313 bp	200 V, 14%, 23.0 °C
	3041–3060	R: GAAATGGCAACCCACTCCAG			
Region-5	8038–8058	F: CACTGGAGATGCGTGGCACAG	62 °C	298 bp	230 V, 14%, 23.7 °C
	8314–8335	R: GAAGCACACAAACATGATGATG			
Region-6	8345–8365	F: AAGCGAGAATTGATGAAGTCG	62 °C	301 bp	200 V, 12%, 23.7 °C
	8624–8645	R: TTCAGCACAGTAGCTTATCTGG			

^a^ The primer binding positions are given relative to GenBank JN604985.1; ^b^ These primers were described previously by Yang et al., 2014 [13].

**Table 2:** (**a**)

Variant	Region-1												
	c.−1846	c.−1760	c.−1738	c.−1710	c.−1678	c.−1645	c.−1644	c.−1640	c.−1636	c.−1624	c.−1569	c.−1538	Frequency ^a^
*A*_1_	C	G	G	G	G	A	G	A	A	T	A	A	86.56%
*B*_1_	C	G	A	G	G	A	G	A	A	T	G	A	3.56%
*C*_1_	C	G	G	A	G	G	A	C	A	C	A	A	1.78%
*D*_1_	C	G	G	G	A	A	G	A	G	T	A	G	2.96%
*E*_1_	T	G	G	G	G	G	A	A	A	T	A	A	0.79%
*F*_1_	C	A	G	A	G	A	A	C	A	C	A	A	4.35%

^a^ Frequency across all nine breeds studied.

**Table 2:** (**b**)

Variant	Region-2		
	c. −1334	c. −1299	Frequency
*A*_2_	C	G	56.35%
*B*_2_	T	G	26.19%
*C*_2_	T	A	17.46%

**Table 2:** (**c**)

Variant	Region-3								
	c.126 + 28	c.126 + 126	c.126 + 127	c.126 + 128	c.126 + 129	c.126 + 205	c.126 + 237	c.126 + 250	Frequency
*A*_3_	A	-	-	G	A	C	A	G	90.23%
*B*_3_	T	A	A	G	A	C	G	G	6.84%
*C*_3_	T	A	A	G	A	C	A	T	0.78%
*D*_3_	A	-	-	-	-	C	A	G	1.56%
*E*_3_	A	-	-	G	A	G	A	G	0.59%

**Table 2:** (**d**)

Variant	Region-5						
	c.804–196	c.804–194	c.804–80	c.804–61	c.804–38	c.804–4	Frequency
*A*_5_	A	A	C	T	C	T	41.30%
*B*_5_	T	G	G	C	T	A	13.05%
*C*_5_	T	G	C	C	T	T	45.65%

**Table 2:** (**e**)

Variant	Region-6		
	c.910 ^a^	c.*9	Frequency
*A*_6_	A	G	50.00%
*B*_6_	G	G	44.27%
*C*_6_	G	A	5.73%

^a^ Non-synonymous.

**Table 3:** Association of ovine *UCP1* haplotypes with carcass muscles trait (Mean ± standard error (SE)) ^a^ in New Zealand (NZ) Romney sheep.

Traits	Haplotype Assessed	*n*		Single-Haplotype Model			Multi-Haplotype Model			
		Present	Absent	Present	Absent	*p* ^b^	Haplotypes Fitted	Present	Absent	*p* ^b^
HCW ^c^ (kg)	*A*_2_-*A*_5_	*86*	*228*	*17.36 ± 0.36*	*17.07 ± 0.32*	*0.189*	*A*_2_-*B*_5_	17.08 *±* 0.26	16.78 *±* 0.21	0.231
	***A*_2_-*B*_5_**	58	256	**16.44 ± 0.40**	**17.21 ± 0.31**	**0.006**	***A*_2_-*A*_5_**	**16.70 ± 0.48**	**17.28 ± 0.33**	**0.017**
	*B*_2_-*A*_5_	175	139	17.12 ± 0.33	17.15 ± 0.33	0.886	*A*_2_-*A*_5_, *A*_2_-*B*_5_	17.19 *±* 0.25	17.07 *±* 0.22	0.597
	*C*_2_-*A*_5_	124	190	17.20 ± 0.33	17.04 ± 0.35	0.492	*A*_2_-*A*_5_, *A*_2_-*B*_5_	17.21 *±* 0.27	17.05 *±* 0.21	0.526
	*C*_2_-*C*_5_	95	219	17.21 ± 0.37	17.11 ± 0.32	0.678	*A*_2_-*A*_5_, *A*_2_-*B*_5_	17.19 *±* 0.28	17.07 *±* 0.20	0.641
V-GR ^d^ (mm)	*A*_2_-*A*_5_	86	228	2.36 ± 0.58	2.13 ± 0.53	0.556				
	*A*_2_-*B*_5_	58	256	1.82 ± 0.66	2.24 ± 0.51	0.367				
	*B*_2_-*A*_5_	175	139	2.05 ± 0.54	2.36 ± 0.54	0.337				
	*C*_2_-*A*_5_	124	190	*2.39 ± 0.53*	*1.89 ± 0.56*	*0.195*				
	*C*_2_-*C*_5_	95	219	2.43 ± 0.59	2.14 ± 0.52	0.452				
Leg lean-meat yield (%)	*A*_2_-*A*_5_	86	228	20.67 ± 0.23	20.84 ± 0.21	0.270				
	*A*_2_-*B*_5_	58	256	20.60 ± 0.26	20.81 ± 0.20	0.243				
	*B*_2_-*A*_5_	175	139	20.83 ± 0.21	20.74 ± 0.21	0.482				
	*C*_2_-*A*_5_	124	190	20.77 ± 0.21	20.82 ± 0.22	0.733				
	*C*_2_-*C*_5_	95	219	*20.63 ± 0.23*	*20.83 ± 0.20*	*0.174*				
Loin lean-meat yield (%)	*A*_2_-*A*_5_	86	228	14.17 ± 0.17	14.22 ± 0.16	0.660				
	***A*_2_-*B*_5_**	58	256	**13.94 ± 0.20**	**14.24 ± 0.15**	**0.032**				
	*B*_2_-*A*_5_	175	139	14.22 ± 0.16	14.18 ± 0.16	0.701				
	*C*_2_-*A*_5_	124	190	14.19 ± 0.16	14.23 ± 0.17	0.730				
	*C*_2_-*C*_5_	95	219	14.21 ± 0.18	14.20 ± 0.15	0.952				
Shoulder lean-meat yield (%)	*A*_2_-*A*_5_	86	228	17.08 ± 0.19	16.97 ± 0.17	0.382				
	*A*_2_-*B*_5_	58	256	16.90 ± 0.22	17.01 ± 0.17	0.475				
	*B*_2_-*A*_5_	175	139	16.96 ± 0.18	17.05 ± 0.18	0.433				
	*C*_2_-*A*_5_	124	190	16.99 ± 0.18	17.02 ± 0.19	0.850				
	*C*_2_-*C*_5_	95	219	17.08 ± 0.20	16.98 ± 0.17	0.458				
Total lean-meat yield (%)	*A*_2_-*A*_5_	86	228	51.92 ± 0.47	52.03 ± 0.43	0.734				
	*A*_2_-*B*_5_	58	256	*51.44 ± 0.53*	*52.06 ± 0.42*	*0.100*				
	*B*_2_-*A*_5_	175	139	52.02 ± 0.44	51.97 ± 0.44	0.872				
	*C*_2_-*A*_5_	124	190	51.95 ± 0.43	52.07 ± 0.46	0.711				
	*C*_2_-*C*_5_	95	219	51.92 ± 0.48	52.02 ± 0.42	0.746				
Proportion leg lean-meat yield (%)	*A*_2_-*A*_5_	86	228	*39.79 ± 0.25*	*40.05 ± 0.22*	*0.122*	*C*_2_-*C*_5_	39.63 *±* 0.25	39.96 *±* 0.23	0.050
	*A*_2_-*B*_5_	58	256	40.05 ± 0.28	39.96 ± 0.22	0.677	*A*_2_-*A*_5_, *C*_2_-*C*_5_	39.92 *±* 0.21	39.85 *±* 0.15	0.738
	*B*_2_-*A*_5_	175	139	40.05 ± 0.23	39.89 ± 0.23	0.261	*A*_2_-*A*_5_, *C*_2_-*C*_5_	39.79 *±* 0.18	39.90 *±* 0.15	0.530
	*C*_2_-*A*_5_	124	190	39.97 ± 0.23	39.98 ± 0.24	0.919	*A*_2_-*A*_5_, *C*_2_-*C*_5_	39.78 *±* 0.20	39.89 *±* 0.15	0.548
	***C*_2_-*C*_5_**	95	219	**39.71 ± 0.25**	**40.03 ± 0.22**	**0.047**	***A*_2_-*A*_5_**	**39.51 ± 0.31**	**39.98 ± 0.24**	**0.022**
Proportion loin lean-meat yield (%)	*A*_2_-*A*_5_	86	228	27.29 ± 0.20	27.32 ± 0.18	0.793				
	*A*_2_-*B*_5_	58	256	*27.07 ± 0.23*	*27.34 ± 0.18*	*0.106*				
	*B*_2_-*A*_5_	175	139	27.34 ± 0.19	27.28 ± 0.19	0.603				
	*C*_2_-*A*_5_	124	190	27.30 ± 0.19	27.32 ± 0.20	0.859				
	*C*_2_-*C*_5_	95	219	27.36 ± 0.21	27.30 ± 0.18	0.615				
Proportion shoulder lean-meat yield (%)	*A*_2_-*A*_5_	86	228	*32.92 ± 0.26*	*32.62 ± 0.23*	*0.085*				
	*A*_2_-*B*_5_	58	256	32.88 ± 0.29	32.70 ± 0.23	0.379				
	*B*_2_-*A*_5_	175	139	*32.61 ± 0.24*	*32.83 ± 0.24*	*0.130*				
	*C*_2_-*A*_5_	124	190	32.73 ± 0.24	32.69 ± 0.25	0.803				
	*C*_2_-*C*_5_	95	219	*32.92 ± 0.26*	*32.67 ± 0.23*	*0.133*				

^a^ Estimated marginal means and standard errors of those means derived from General Linear Mixed Models (GLMMs). ^b^ *p* < 0.05 in bold, and 0.05 ≤ *p* < 0.20 in italic. ^c^ HCW represents Hot Carcass Weight. ^d^ V-GR represents VIAScan fat score.
